# Transcutaneous Vagus Nerve Stimulation (tVNS) does not increase prosocial behavior in Cyberball

**DOI:** 10.3389/fpsyg.2015.00499

**Published:** 2015-04-28

**Authors:** Roberta Sellaro, Laura Steenbergen, Bart Verkuil, Marinus H. van IJzendoorn, Lorenza S. Colzato

**Affiliations:** ^1^Cognitive Psychology Unit, Institute for Psychological Research, Leiden UniversityLeiden, Netherlands; ^2^Leiden Institute for Brain and Cognition, Leiden UniversityLeiden, Netherlands; ^3^Centre for Child and Family Studies, Leiden UniversityLeiden, Netherlands

**Keywords:** Vagus Nerve Stimulation, insula, Cyberball, vicarious ostracism, PFC

## Abstract

Emerging research suggests that individuals experience vicarious social pain (i.e., ostracism). It has been proposed that observing ostracism increases activity in the insula and in the prefrontal cortex (PFC), two key brain regions activated by directly experiencing ostracism. Here, we assessed the causal role of the insula and PFC in modulating neural activity in these areas by applying transcutaneous Vagus Nerve Stimulation (tVNS), a new non-invasive and safe method to stimulate the vagus nerve that has been shown to activate the insula and PFC. A single-blind, sham-controlled, within-subjects design was used to assess the effect of on-line (i.e., stimulation overlapping with the critical task) tVNS in healthy young volunteers (*n* = 24) on the prosocial Cyberball game, a virtual ball-tossing game designed to measure prosocial compensation of ostracism. Active tVNS did not increase prosocial helping behavior toward an ostracized person, as compared to sham (placebo) stimulation. Corroborated by Bayesian inference, we conclude that tVNS does not modulate reactions to vicarious ostracism, as indexed by performance in a Cyberball game.

## Introduction

People vicariously experience others’ (social) pain. Several recent studies have demonstrated vicarious ostracism (i.e., the observation of other people being socially ignored and excluded). These studies show that spectators identify with an ostracized individual’s pain and also feel ostracized themselves (Over and Carpenter, 2009; Wesselmann et al., 2009; Masten et al., 2010, 2011a, 2013a,b; Beeney et al., 2011; Meyer et al., 2012; Will et al., 2013). As pointed out by Wesselmann et al. (2013a), not only adults (Wesselmann et al., 2009; Beeney et al., 2011; Masten et al., 2011a; Meyer et al., 2012; Will et al., 2013) but also children and adolescents (Over and Carpenter, 2009; Masten et al., 2010, 2013a,b; Will et al., 2013) exhibit vicarious ostracism.

In the literature, a reliable index of vicarious ostracism is an adapted version of the Cyberball game (Williams, 2009), a virtual ball-tossing game in which participants observe someone else being ostracized. Perceiving someone being ostracized during the Cyberball game presents the participant with a moral conflict: helping the ostracized person by throwing the ball to the victim more often, or following the other computer-controlled confederates by excluding the victim (Williams and Jarvis, 2006). Using this version of the Cyberball game, previous research has shown that people typically tend to compensate for other individuals’ ostracism by throwing the ball toward the ostracized person more often (Riem et al., 2013; Wesselmann et al., 2013b), unless they are induced to think that the ostracized individual deserved this treatment (Wesselmann et al., 2013b). Interestingly, observing ostracism increases activity in the insula and anterior cingulate cortex, the key social pain-related regions that are activated also by directly experiencing ostracism (Eisenberger and Lieberman, 2004). Moreover, observing ostracism activates the prefrontal cortex (PFC) and precuneus—brain regions associated with mentalization (i.e., ability to understand the mental state of oneself and others; Masten et al., 2010, 2011a,b, 2013b). Brain activation of both the mentalization areas and social pain-related regions correlates with individual differences in empathy when observing ostracism and with prosocial behavior toward the victim, which has been taken to suggest that differences in experiencing vicarious ostracism may also reflect individual differences in trait empathy (Masten et al., 2010, 2011a,b, 2013a).

Here, we assessed the causal role of this PFC-insula network in mediating vicarious ostracism by applying transcutaneous Vagus Nerve Stimulation (tVNS), a new non-invasive and safe method to stimulate the vagus nerve, introduced for the first time by Ventureyra (2000; for a recent review see Vonck et al., 2014). tVNS stimulates the afferent auricular branch of the vagus nerve located medial of the tragus at the entry of the acoustic meatus (Kreuzer et al., 2012). tVNS is safe and is accompanied only with minor side effects such as an itching sensation under the electrodes. Very recently, it has been suggested that tVNS may be a valuable tool for modulating cognitive processes in healthy humans (van Leusden et al., 2015). Two functional magnetic resonance imaging (MRI) studies in healthy humans have shown increased activation during active tVNS in the locus coeruleus and the solitary tract, as an indication of effective stimulation of the vagal afferences and both the insula and PFC (Dietrich et al., 2008; Kraus et al., 2013), which are key areas related to social pain and mentalization, and linked to vicarious ostracism.

Given the available correlational evidence that vicarious ostracism involves the PFC-insula network, we tested whether tVNS enhances prosocial helping behavior toward an ostracized person who was unknown to the participant. This hypothesis is supported by the findings that tVNS produces a reliable activation in both the insula and the PFC (Dietrich et al., 2008; Kraus et al., 2013). Accordingly, we assessed the effect of on-line (i.e., stimulation overlapping with the critical task) tVNS on an adapted version of the Cyberball game to measure compensation for other players’ ostracism. A positive effect of tVNS during Cyberball would be indicated by an increased number of tosses toward the ostracized person.

## Experimental Procedures

### Participants

Twenty-four Leiden University undergraduate students (21 females, three males, mean age = 19.13 years, range 18–22) participated in the experiment. Participants were recruited via an on-line recruiting system and were offered course credit for participating in a study on the effects of brain stimulation on social decision-making. Participants were screened individually via a phone interview by the same lab-assistant using the Mini International Neuropsychiatric Interview (M.I.N.I.). The M.I.N.I. is a short, structured interview of about 15 min that screens for several psychiatric disorders and drug use, often used in clinical and pharmacological research (Sheehan et al., 1998; Colzato and Hommel, 2008; Colzato et al., 2009). Participants were considered suitable to participate in this study if they fulfilled the following criteria: (i) age between 18 and 30 years; (ii) no history of neurological or psychiatric disorders; (iii) no history of substance abuse or dependence; (iv) no history of brain surgery, tumors, or intracranial metal implantation; (v) no chronic or acute medications; (vi) no pregnancy; (vii) no susceptibility to seizures or migraine; (viii) no pacemaker or other implanted devices.

All participants were naïve to tVNS. Prior to the testing session, they received a verbal and written explanation of the procedure and of the typical adverse effects (i.e., itching and tingling skin sensation, skin reddening, and headache). No information was provided about the different types of stimulation (active vs. sham) or about the hypotheses concerning the experiment. The study conformed to the ethical standards of the Declaration of Helsinki and the protocol was approved by the medical ethics committee (Leiden University Medical Center).

### Apparatus and Procedure

A single-blinded, sham/placebo-controlled, randomized cross-over within-subjects study with counterbalanced order of conditions was used to assess the effect of on-line (i.e., stimulation overlapping with the critical task) tVNS on a prosocial Cyberball game in healthy young volunteers.

All participants took part in two sessions (active vs. sham) and were tested individually. In both sessions, upon arrival, participants were asked to rate their mood on a 9 × 9 Pleasure × Arousal grid (Russell et al., 1989) with values ranging from -4 to 4. Heart rate (HR) and systolic and diastolic blood pressure (SBP and DBP) were collected from the non-dominant arm with an OSZ 3 Automatic Digital Electronic Wrist Blood Pressure Monitor (Speidel & Keller) for the first time (T1). Immediately after, participants performed either the Empathy Quotient (EQ; in session 1) or the interpersonal reactivity index (IRI; in session 2). The EQ is a self-report questionnaire designed to assess empathy in normal adult populations (Cronbach’s alpha is 0.92; Baron-Cohen and Wheelwright, 2004). It comprises 60 questions (20 items are filler questions) that, taken together, provide an overall measure of cognitive perspective taking, affective empathy, and social skills (range 0–80, higher scores = more empathy). The IRI is a self-report questionnaire that assesses perceived individual differences in the tendency to be empathetic. It consists of 28 Likert-type items on a response scale with five alternatives ranging from 0 (Does not describe me well) to 4 (Describes me very well). It comprises four subscales assessing affective (empathic concern and personal distress) and cognitive (fantasy and perspective taking) components of empathy (Davis, 1980, 1983). Cronbach’ s alphas are 0.73, 0.77, 0.83, and 0.73 for the emphatic concern, personal distress, fantasy, and perspective taking subscales, respectively (De Corte et al., 2007). Afterwards, participants rated again their mood and HR, SBP, and DBP were collected for the second time (T2). Then, they performed for 8 min each two unrelated computer tasks tapping into emotional working memory and implicit biased attitudes (data not reported here) before rating their mood and having HR, SBP, and DBP measured for the third time (T3). After that, participants performed the prosocial Cyberball game, which lasted for 10 min. Once completed the Cyberball, mood, HR, SBP, and DBP were measured for the fourth time (T4). tVNS was applied throughout all three computer tasks.

#### Transcutaneous Vagus Nerve Stimulation (tVNS)

We used a tVNS wired neurostimulating device connected with two titan electrodes fastened on a gel frame (CM02, Cerbomed, Erlangen, Germany). Following the suggestions by Dietrich et al. (2008) and Steenbergen et al. (2015) for optimal stimulation, the tVNS^®^device was programmed to a stimulus intensity at 0.5 mA, delivered with a pulse width of 200–300 μs at 25 Hz. Stimulation alternated between On/Off periods every 30 s. In the sham (placebo) condition, the stimulation electrodes were placed on the center of the left ear lobe instead of the outer auditory canal. Indeed, the ear lobe has been found to be free of cutaneous vagal innervation (Peuker and Filler, 2002; Fallgatter et al., 2003) and a recent fMRI study showed that this sham condition produced no activation in the cortex and brain stem (Kraus et al., 2013).

Importantly, following safety criteria to avoid cardiac side effects, the stimulation was always applied to the left ear (Nemeroff et al., 2006; Cristancho et al., 2011). Indeed, although efferent fibers of the vagus nerve modulate cardiac function, such a modulation seems to relate only to the efferent vagal fibers connected to the right ear (Nemeroff et al., 2006). Consistently, a clinical trial showed no arrhythmic effects of tVNS when applied to the left ear (Kreuzer et al., 2012).

#### Prosocial Cyberball

**FIGURE 1:**
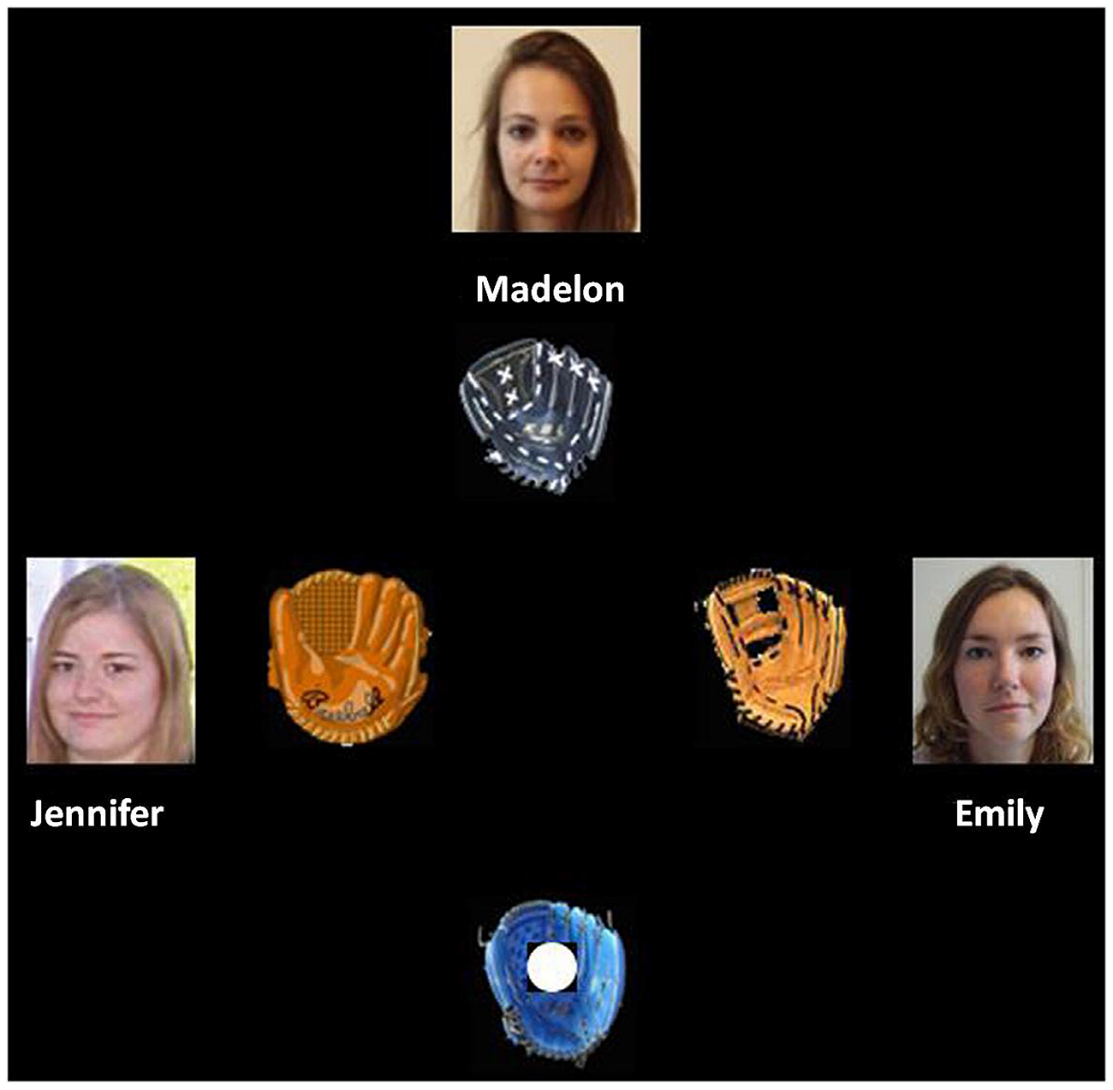
Set-up Cyberball task in the neutral condition. The participants’ glove was at the bottom of the screen. The glove, name, and picture of the unknown victim with a neutral or sad expression were at the upper part of the screen. The gloves, names, and pictures of the other unknown players were to the left and right of the screen center. Figure taken from Riem et al. (2013).

The Cyberball game was an adapted version of the task used in the study by Riem et al. (2013). The game was a virtual online group interaction involving four players throwing a ball to each other. Participants were led to believe that they would play this game via Internet with three other unknown peers. In reality, the other players were virtual computer-controlled confederates. The participants’ glove was at the bottom of the screen. The gloves, names, and pictures of the unknown victim and of the other two unknown players were displayed in the upper part of the screen, and to the left and to right of the screen, respectively (see **Figure 1**). A computer keyboard was used by the participants to throw the ball to the other players.

The game consisted of two parts with a short break in between, each comprising three 48-trial blocks. The first block was programmed to create a fair situation where all players received the ball equally often (i.e., fair play block). The second (i.e., unfair play block 1) and the third (i.e., unfair play block 2) blocks were programmed to establish an unfair situation in which one of the players (i.e., the victim) never received any throw from the two unknown players. The third block included an additional manipulation: the facial expression of the ostracized player changed from neutral to sad (i.e., unfair play block 2 with sad victim), or remained neutral (i.e., unfair play block 2 with neutral victim). Half of the participants were confronted with the ostracized player showing a sad expression in the third block of the first part, and with the ostracized player showing a neutral expression in the third block of the second part. The remaining participants experienced the two conditions in the reversed order. The sad facial expression did not change when the participant threw the ball to the ostracized victim.

The dependent variable was the number of ball tossing to the victim, calculated as the ratio between the number of throws of the participant to the victim and the total number of throws by the participant to any of the players. Ratios were calculated for each play block. A ratio larger than 0.33 in the unfair play block indicates that participants compensate for the other player’ ostracism by throwing the ball to the victim more often.

### Statistical Analyses

To examine whether active tVNS, as compared to sham (placebo) stimulation, influenced prosocial behavior, as indexed by the number of tossing to the ostracized player, repeated-measures analysis of variance (ANOVA) was carried out with the ratio of ball throws to the victim as dependent variable and play block (fair play blocks, unfair play block 1, unfair play block 2 with neutral victim, unfair play block 2 with sad victim) and session (active vs. sham) as within-participants factors. Mood (i.e., pleasure and arousal scores), HR, SBP, and DBP were analyzed separately by means of repeated-measures ANOVAs with effect of time (first vs. second vs. third vs. fourth measurement) and session (active vs. sham) as within-participants factors.

A significance level of *p* < 0.05 was adopted for all statistical tests. Tukey HSD *post hoc* tests were performed to clarify mean differences.

Furthermore, we calculated Bayesian (posterior) probabilities associated with the occurrence of the null [*p*(H_0_| D)] and alternative [*p*(H_1_| D)] hypotheses, given the observed data. Bayesian analyses allow making inferences about both significant and non-significant effects by estimating the probability of their occurrence, with values ranging from 0 (i.e., no evidence) to 1 (i.e., very strong evidence; see Raftery, 1995). To calculate Bayesian probabilities we used the method proposed by Wagenmakers (2007) and Masson (2011). This method uses Bayesian information criteria (BIC), calculated using a simple transformation of sum-of-squares values generated by the standard ANOVA, to estimate Bayes factors and generate *p*(H_0_| D) and *p*(H_1_| D), assuming a “unit information prior” (for further details, see Kass and Wasserman, 1995; see also Jarosz and Wiley, 2014).

## Results

### Cyberball Task

**FIGURE 2:**
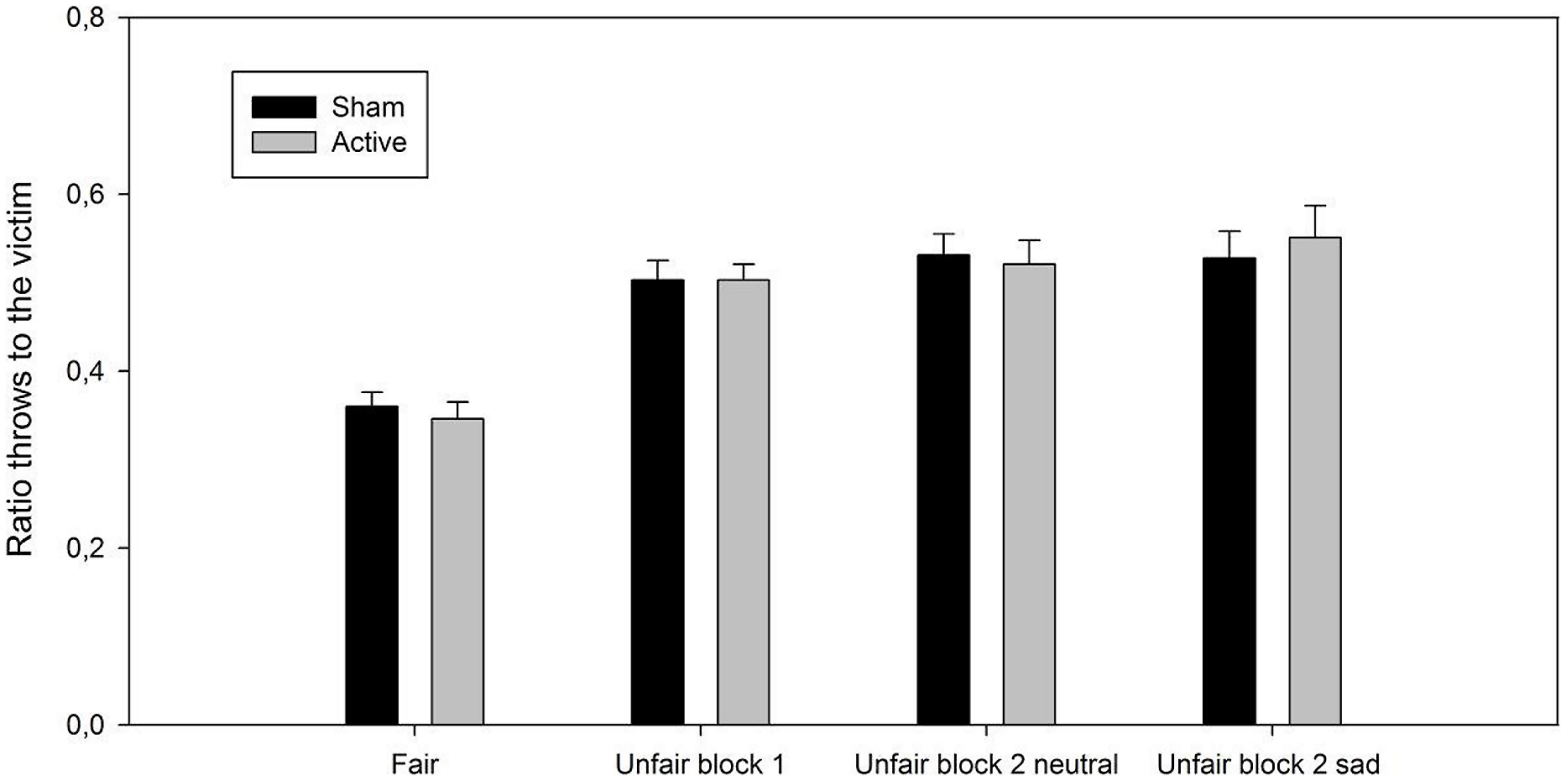
Ratio of throws (M, SEM) to the excluded player as a function of play block (fair play block, unfair play block 1, and unfair block 2 with the neutral and sad victim) and session (active and sham).

ANOVA revealed a significant effect of play block [*F*(3,69) = 29.58, *p*< 0.001, $\eta_{p}^{2}$ = 0.56, *p*(H_1_| D) = 0.83]. *Post hoc* tests showed that participants threw the ball more often to the victim in the unfair blocks compared to the fair block (*p*_s_ < 0.001, Cohen’s *d*_s_ ≥ 1.45). There were no significant differences between the three types of unfair blocks (*p*_s_ ≥ 0.36, Cohen’s *d*_s_ ≤ 0.27). Importantly, neither the main effect of session [*F*(1,23) < 1, *p*= 0.99, $\eta_{p}^{2}$ < 0.001, *p*(H_0_| D) > 0.99] nor the session by play block interaction [*F*(3,69) < 1, *p*= 0.76, $\eta_{p}^{2}$ = 0.02, *p*(H_0_| D) > 0.99] reached statistical significance, see **Figure 2**.

### Empathy Quotient (EQ) and Interpersonal Reactivity Index (IRI)

For both the EQ and IRI, participants’ scores were comparable to the values typically observed in healthy participants: EQ (47.96, SD = 9.8); IRI_total score_ (66.75, SD = 12.11); IRI_perspective taking_ (18.42, SD = 4.8); IRI_fantasy scale_ (16.79, SD = 5.8); IRI_emphatic concern_ (18.79, SD = 4.0); IRI_personal_ _distress_ (12.75, SD = 3.3). In order to examine the possible role of individual differences in empathy, Pearson correlations coefficients were computed between the ratio of ball throws to the victim and participants’ EQ and IRI scores, separately for the four blocks (fair play blocks, unfair play block 1, unfair play block 2 with neutral victim, unfair play block 2 with sad victim) and the two sessions (active and sham). No significant correlations were observed (*p*_s_ ≥ 0.07).

### Physiological and Mood Measurements

**Table 1** provides an overview of the outcomes for physiological and mood measurements. ANOVAs showed a main effect of timing for pleasure [*F*(3,69) = 4.15, *p* = 0.009, $\eta_{p}^{2}$ = 0.15, but *p*(H_1_| D) = 0.39], but not for the other variables (*F*_s_ ≤ 1.0, *p*_s_ ≥ 0.39, η_ps_^2^ ≤ 0.04, *p*_s_(H_0_| D) ≥ 0.99). Pleasure levels dropped at the third measurement and rose again at the fourth one (1.5 vs. 1.5 vs. 1.2 vs. 1.5). Indeed, *post hoc* tests revealed that pleasure levels at the third measurement were significantly different from levels at the first, second, and forth measurements (*p*_s_ ≤ 0.05, Cohen’s *d*_s_ ≥ 0.42). No significant differences were observed when comparing scores at the first, second, and forth measurements to each other (*p*_s_ ≥ 0.99, Cohen’s *d*_s_ ≤ 0.11). Importantly, HR, DBP, SBP, pleasure, and arousal did not significantly differ between the two sessions. Indeed, neither the main effects of session nor the two-way interactions involving session and time were significant [*F*_s_ ≤ 1.76, *p*_s_ ≥ 0.16, η_ps_^2^ ≤ 0.07, *p*_s_(H_0_| D) ≥ 0.71]. Significant differences between the two sessions were not observed either when considering only participants’ scores measured immediately before (T3) and at the end of the Cyberball game (T4) [*F*_s_ ≤ 2.7, *p*_s_ ≥ 0.12, η_ps_^2^ ≤ 0.11, *p*_s_(H_0_| D) ≥ 0.60].

**Table 1:** Mean heart rate (HR) values (in beats per minute), systolic and diastolic blood pressure (SBP and DBP; in mmHg), and arousal and pleasure scores as function of effect of time [first (T1) vs. second (T2) vs. third (T3) vs. forth (T4) measurement; see text for more details] for active and sham (placebo) sessions.

	T1		T2		T3		T4	
	Active	Sham	Active	Sham	Active	Sham	Active	Sham
HR	79.9 (2.8)	81.5 (2.7)	82.4 (3.0)	76.1 (1.8)	78.6 (2.6)	79.4 (4.2)	79.8 (2.8)	74.0 (2.3)
SBP	118.0 (3.1)	118.5 (3.3)	116.7 (3.0)	114.0 (2.8)	118.8 (2.6)	117.2 (3.0)	116.3 (3.1)	118.8 (2.8)
DBP	70.4 (2.1)	72.1 (2.1)	72.9 (2.1)	72.6 (2.8)	72.8 (1.8)	70.0 (1.6)	71.4 (1.8)	72.5 (2.1)
Arousal	0.8 (0.3)	0.7 (0.2)	0.5 (0.3)	0.8 (0.2)	0.4 (0.3)	0.7 (0.3)	0.4 (0.3)	0.5 (0.3)
Pleasure	1.5 (0.2)	1.5 (0.2)	1.6 (0.2)	1.5 (0.2)	1.3 (0.2)	1.0 (0.3)	1.5 (0.2)	1.5 (0.2)

*SEM are shown in parentheses*.

## Discussion

Our results, corroborated by Bayesian inference, suggest that tVNS does not directly modulate reactions to vicarious ostracism in a Cyberball game: participants did not throw more balls toward the unknown ostracized person in the active stimulation compared to sham (placebo). Given that the insula and the PFC seem to be involved in vicarious ostracism (Masten et al., 2011a, 2013a) and that tVNS produces a reliable activation in both the insula and the PFC (Dietrich et al., 2008; Kraus et al., 2013), we expected active tVNS to enhance prosocial helping behavior, leading participants to increase their tendency to compensate the victim for the other players’ ostracism. We can only speculate what the reasons for this outcome pattern are. First, we considered just one index of vicarious ostracism. Even though this index is frequently used and well-established, it remains to be seen whether other measurements of vicarious ostracism can be affected by tVNS. In our current study the victim was unknown to the participant, and an interesting example to consider would be to use a Cyberball game in which the ostracized player is known to the participant and/or to manipulate the group membership (in-group vs. out-group) of the victim. That being said, it is possible that the version of the task we used was not sensitive enough to allow possible tVNS-induced modulations to be detected. Second, and related to the previous point, the lack of a tVNS modulation on vicarious ostracism may be related to the sample of participants tested in the current study, who showed high empathy. As mentioned in the introduction, compensatory behavior following vicarious ostracism is reckoned to reflect trait empathy (Masten et al., 2010). Indeed, people high in trait empathy tend to experience augmented vicarious ostracism and show higher activation in empathy-related brain regions, that is, in the same regions that are activated when observing ostracism (Masten et al., 2010, 2011a,b, 2013a) and that were targeted by tVNS stimulation. Thus, it is plausible that tVNS was not effective at modulating reactions to vicarious ostracism because participants already displayed a lot of empathy (i.e., hitting a ceiling effect), which prevented a possible tVNS-induced effect from emerging. This may also explain why we failed to observe any significant correlation between trait empathy and compensatory behavior. Furthermore, individual differences in family background may at least partially account for the lack of effectiveness of our manipulation. For instance, in a previous study applying intranasal oxytocin, behavioral effects were only found in participants with rather warm relationships with their parents (Riem et al., 2013), and similar neural effects moderated by childhood experiences have also been suggested (Bakermans-Kranenburg and Van IJzendoorn, 2013). Thus, it would be crucial for follow-up studies to assess the role of past experiences and/or the quality of early relationships in moderating the possible effectiveness of tVNS in promoting prosocial behavior. Third, in our study we used a current of 0.5 mA. While this intensity was sufficient to enhance response selection (Steenbergen et al., 2015), changing vicarious ostracism may require greater intensities.

Finally, there are some limitations of the current study that warrant discussion. First, it would have been optimal to have linked the implementation of tVNS with appropriate physiological assays, such as the vagus-evoked potentials (see Bestmann et al., 2014, for a related discussion). Follow-up studies might consider a more thorough exploration of vicarious ostracism through scalp-EEG measures, such as P3b component and frontal EEG asymmetry, two cortical correlates of ostracism (Kawamoto et al., 2013). Second, we did not explicitly assess participants’ blinding by asking them if they could guess the stimulation received.

In sum, we failed to obtain any evidence that tVNS, by increasing insula and PFC neural activity, is effective at modulating reactions to vicarious ostracism in a Cyberball game. Notwithstanding, our results may be useful. First, they can inform future studies on how to better design tVNS experiments to possibly affect vicarious ostracism and prosocial compensation and, second, to suggest potential future directions in this field.

## Conflict of Interest Statement

The authors declare that the research was conducted in the absence of any commercial or financial relationships that could be construed as a potential conflict of interest.

## References

[B1] Bakermans-Kranenburg M. J. Van IJzendoorn M. H. (2013). Sniffing around oxytocin: review and meta-analyses of trials in healthy and clinical groups with implications for pharmacotherapy. *Transl. Psychiatry* 3:e258 10.1038/tp.2013.34 PMC3669921 23695233

[B2] Baron-Cohen S. Wheelwright S. (2004). The empathy quotient: an investigation of adults with Asperger syndrome or high functioning autism, and normal sex differences. *J. Autism Dev. Disord.* 34 163–175 10.1023/b:jadd.0000022607.19833.00 15162935

[B3] Beeney J. E. Franklin R. G. Levy K. N. Adams R. B. (2011). I feel your pain:emotional closeness modulates neural responses to empathically experienced rejection. *Soc. Neurosci.* 6 369–376 10.1080/17470919.2011.557245 21400358

[B4] Bestmann S. de Berker A. O. Bonaiuto J. (2014). Understanding the behavioural consequences of noninvasive brain stimulation. *Trends Cogn. Sci.* 19 13–20 10.1016/j.tics.2014.10.003 25467129

[B5] Colzato L. S. Hommel B. (2008). Cannabis, cocaine, and visuomotor integration: evidence for a role of dopamine D1 receptors in binding perception and action. *Neuropsychologia* 46 1570–1575 10.1016/j.neuropsychologia.2007.12.014 18242650

[B6] Colzato L. S. Slagter H. A. van den Wildenberg W. P. M. Hommel B. (2009). Closing one’s eyes to reality: evidence for a dopaminergic basis of psychoticism from spontaneous eye blink rates. *Pers. Indiv. Differ.* 46 377–380 10.1016/j.paid.2008.10.017

[B7] Cristancho P. Cristancho M. A. Baltuch G. H. Thase M. E. O’Reardon J. P. (2011). Effectiveness and safety of vagus nerve stimulation for severe treatment-resistant major depression in clinical practice after FDA approval: outcomes at 1 year. *J. Clin. Psychiatry* 72 1376–1382 10.4088/jcp.09m05888blu 21295002

[B8] Davis M. H. (1980). A multidimensional approach to individual differences in empathy. *JSAS Catalog Sel. Doc. Psychol.* 10 85.

[B9] Davis M. H. (1983). Measuring individual differences in empathy: evidence for a multidimensional approach. *J. Pers. Soc. Psychol.* 44 113–126 10.1037//0022-3514.44.1.113

[B10] De Corte K. Buysse A. Verhofstadt L. L. Roeyers H. Ponnet K. Davis M. H. (2007). Measuring empathic tendencies: reliability and validity of the Dutch version of the interpersonal reactivity index. *Psychol. Belg.* 47 235–260 10.5334/pb-47-4-235

[B11] Dietrich S. Smith J. Scherzinger C. Hofmann-Preiss K. Freitag T. Eisenkolb A. (2008). A novel transcutaneous vagus nerve stimulation leads to brainstem and cerebral activations measured by functional MRI. *Biomed. Tech.* 53 104–111 10.1515/bmt.2008.022 18601618

[B12] Eisenberger N. I. Lieberman M. D. (2004). Why rejection hurts: the neuro-cognitive overlap between physical and social pain. *Trends Cogn. Sci.* 8 294–300 10.1016/j.tics.2004.05.010 15242688

[B13] Fallgatter A. J. Neuhauser B. Herrmann M. J. Ehlis A. C. Wagener A. Scheuerpflug P. (2003). Far field potentials from the brain stem after transcutaneous vagus nerve stimulation. *J. Neural Transm.* 110 1437–1443 10.1007/s00702-003-0087-6 14666414

[B14] Jarosz A. F. Wiley J. (2014). What are the odds? A practical guide to computing and reporting Bayes Factors. *J. Probl. Solving* 7:2 10.7771/1932-6246.1167

[B15] Kass R. E. Wasserman L. (1995). A reference Bayesian test for nested hypotheses and its relationship to the Schwarz criterion. *J. Am. Stat. Assoc.* 90 928–934 10.1080/01621459.1995.10476592

[B16] Kawamoto T. Nittono H. Ura M. (2013). Cognitive, affective, and motivational changes during ostracism: an ERP, EMG, and EEG study using a computerized cyberball task. *Neurosci. J.* 2013:304674 10.1155/2013/304674 PMC4437265 26317090

[B17] Kraus T. Kiess O. Hösl K. Terekhin P. Kornhuber J. Forster C. (2013). CNS BOLD fMRI effects of sham-controlled transcutaneous electric nerve stimulation in the left outer auditory canal – a pilot study. *Brain Stimul.* 6 798–804 10.1016/j.brs.2013.01.011 23453934

[B18] Kreuzer P. M. Landgrebe M. Husser O. Resch M. Schecklmann M. Geisreiter F. (2012). Transcutaneous vagus nerve stimulation: retrospective assessment of cardiac safety in a pilot study. *Front. Psychiatry* 3:70 10.3389/fpsyt.2012.00070 PMC3413045 22891061

[B19] Masson M. E. J. (2011). A tutorial on a practical Bayesian alternative to null hypothesis significance testing. *Behav. Res. Methods* 43 679–690 10.3758/s13428-010-0049-5 21302025

[B20] Masten C. L. Eisenberger N. I. Pfeifer J. H. Colich N. L. Dapretto M. (2013a). Associations among pubertal development, empathicability, and neural responses while witnessing peer rejectionin adolescence. *Child Dev*. 84 1338–1354 10.1111/cdev.12056 23379360 PMC3659192

[B21] Masten C. L. Eisenberger N. I. Pfeifer J. H. Dapretto M. (2013b). Neural responses to witessing peer rejection after being socially excluded: fMRI as a window into adolescents’emotional processing. *Dev. Sci.* 16 743–759 10.1111/desc.12056 24033579 PMC3775008

[B22] Masten C. L. Eisenberger N. I. Pfeifer J. H. Dapretto M. (2010). Witnessing peer rejection during early adolescence: neural correlates of empathy for experiences of social exclusion. *Soc. Neurosci.* 5 496–507 10.1080/17470919.2010.490673 20602283 PMC2957502

[B23] Masten C. L. Morelli S. A. Eisenberger N. I. (2011a). An fMRI investigation of empathy for ‘social pain’ and subsequent prosocial behavior. *Neuroimage* 55 381–388 10.1016/j.neuroimage.2010.11.060 21122817

[B24] Masten C. L. Telzer E. H. Eisenberger N. I. (2011b). An fMRI investigation of attributing negative social treatment to racial discrimination. *J. Cogn. Neurosci.* 23 1042–1051 10.1162/jocn.2010.21520 20521861

[B25] Meyer M. L. Masten C. L. Ma Y. Wang C. Shi Z. Eisenberger N. I. (2012). Empathy for the social suffering of friends and strangers recruits distinct patterns of brain activation. *Soc. Cogn. Affect. Neurosci.* 8 446–454 10.1093/scan/nss019 22355182 PMC3624958

[B26] Nemeroff C. B. Mayberg H. S. Krahl S. E. Mcnamara J. Frazer A. Henry T. R. (2006). VNS therapy in treatment- resistant depression: clinical evidence and putative neurobiological mechanisms. *Neuropsychopharmacology* 31 1345–1355 10.1038/sj.npp.1301190 16641939

[B27] Over H. Carpenter M. (2009). Priming third-party ostracism increases affiliative imitation in children. *Dev. Sci.* 12 F1–F8 10.1111/j.1467-7687.2008.00820.x 19371357

[B28] Peuker E. T. Filler T. J. (2002). The nerve supply of the human auricle. *Clin. Anat.* 15 35–37 10.1002/ca.1089 11835542

[B29] Raftery A. E. (1995). “Bayesian model selection in social research,” in *Sociological Methodology*, ed. Marsden P. V. (Oxford: Blackwells), 111–196 10.2307/271063

[B30] Riem M. M. Bakermans-Kranenburg M. J. Huffmeijer R. van IJzendoorn M. H. (2013). Does intranasal oxytocin promote prosocial behavior to an excluded fellow player? a randomized-controlled trial with Cyberball. *Psychoneuroendocrinology* 38 1418–1425 10.1016/j.psyneuen.2012.12.023 23352229

[B31] Russell J. A. Weis A. Mendelsohn G. A. (1989). Affect grid: a single-item scale of pleasure and arousal. *J. Pers. Soc. Psychol.* 57 493–502 10.1037/0022-3514.57.3.493

[B32] Sheehan D. V. Lecrubier Y. Sheehan K. H. Amorim P. Janavs J. Weiller E. (1998). The Mini-International Neuropsychiatric Interview (M.I.N.I.): the development and validation of a structured diagnostic psychiatric interview for DSM-IV and ICD-10. *J. Clin. Psychiatry* 59 22–23. 9881538

[B33] Steenbergen L. Sellaro R. Stock A.-K. Verkuil B. Beste C. Colzato L. S. (2015). Transcutaneous vagus nerve stimulation (tVNS) enhances response selection during action cascading processes. *Eur. Neuropsychopharm.* (in press). 10.1016/j.euroneuro.2015.03.015 25869158

[B34] van Leusden J. W. R. Sellaro R. Colzato L. S. (2015). Transcutaneous vagal nerve stimulation (tVNS): a new neuromodulation tool in healthy humans? *Front. Psychol.* 6:102 10.3389/fpsyg.2015.00102 PMC4322601 25713547

[B35] Ventureyra E. C. (2000). Transcutaneous vagus nerve stimulation for partial onset seizure therapy. *Child. Nerv. Syst.* 16 101–102 10.1007/s003810050021 10663816

[B36] Vonck K. Raedt R. Naulaerts J. De Vogelaere F. Thiery E. Van Roost D. (2014). Vagus nerve stimulation… 25 years later! What do we know about the effects on cognition? *Neurosci. Biobehav. Rev.* 45 63–71 10.1016/j.neubiorev.2014.05.005 24858008

[B37] Wagenmakers E.-J. (2007). A practical solution to the pervasive problems of p values. *Psychon. Bull. Rev.* 14 779–804 10.3758/BF03194105 18087943

[B38] Wesselmann E. D. Bagg D. Williams K. D. (2009). “I feel your pain”: the effects of observing ostracism on the ostracism detection system. *J. Exp. Soc. Psychol.* 45 1308–1311 10.1016/j.jesp.2009.08.003

[B39] Wesselmann E. D. Williams K. D. Hales A. H. (2013a). Vicarious ostracism. *Front. Hum. Neurosci.* 7:153 10.3389/fnhum.2013.00153 PMC3632775 23630484

[B40] Wesselmann E. D. Wirth J. H. Pryor J. B. Reeder G. D. Williams K. D. (2013b). When do we ostracize? *Soc. Psychol. Pers. Sci.* 4 108–115 10.1177/1948550612443386

[B41] Will G.-J. Crone E. A. Van den Bos W. Güroglu B. (2013). Acting on observed social exclusion: developmental perspectives on punishment of excluders and compensation of victims. *Dev. Psychol.* 49 2236–2244 10.1037/a0032299 23544860

[B42] Williams K. D. (2009). “Ostracism: effects of being excluded and ignored,” in *Advances in Experimental Social Psychology*, ed. Zanna M. (NewYork, NY: Academic Press), 275–314 10.4135/9781412958479.n384

[B43] Williams K. D. Jarvis B. (2006). Cyberball: a program for use in research on interpersonal ostracism and acceptance. *Behav. Res. Methods* 38 174–180 10.3758/bf03192765 16817529

